# Characteristics, incidence and outcome of polymicrobial bloodstream infections: a nationwide population-based study, Finland, 2004–2018

**DOI:** 10.1007/s15010-025-02642-5

**Published:** 2025-09-25

**Authors:** Keiju S.K. Kontula, Kirsi Skogberg, Jukka Ollgren, Asko Järvinen, Outi Lyytikäinen

**Affiliations:** 1 Division of Infectious Diseases, Inflammation Center, Helsinki University Hospital and University of Helsinki, P.O. Box 340, Helsinki, 00029 HUS Finland; 2 Department of Public Health, Finnish Institute for Health and Welfare, P.O. Box 30, Helsinki, FI-00271 Finland

**Keywords:** Bloodstream infection, Comorbidity, Incidence, Mortality

## Abstract

**Purpose:**

Bloodstream infections (BSI) are associated with high mortality. Previous studies have reported worse outcome for polymicrobial than for monomicrobial BSIs. We analyzed patient characteristics and temporal trends of the incidence and outcome of polymicrobial BSIs in Finland during 2004–2018.

**Methods:**

We used data from national registries to identify polymicrobial BSIs during 2004–2018 and to determine origin of infection, patients’ comorbidities and death within 30 days. Charlson comorbidity index (CCI) was calculated according to ICD-10 diagnose codes.

**Results:**

In total, 173,715 BSIs were identified; 11,347 (6.5%) were polymicrobial. Compared with monomicrobial BSIs, the proportion of males, healthcare-associated BSIs, and patients with high CCI were greater in polymicrobial BSIs (58.5% vs. 51.5%, 34.7% vs. 28.7%, and 24.9% vs. 21.1%, respectively). *Escherichia coli*, enterococci, coagulase-negative staphylococci, and *Klebsiella* sp. were the most common pathogens of polymicrobial BSIs. Anaerobic bacteria were noted in 16.3% of polymicrobial BSIs, compared with 4.3% of monomicrobial BSIs. The annual polymicrobial BSI incidence rose from 9.7 to 21.8/100,000 population during 2004–2018, most sharply among patients aged ≥ 90 years. The 30-day case fatality of polymicrobial BSIs was 20.6%, significantly higher than in monomicrobial BSIs (12.4%), and a decline from 25.2 to 20.8% was observed over time.

**Conclusion:**

Polymicrobial BSI incidence increased twofold during 2004–2018. The case fatality was considerably higher in polymicrobial than in monomicrobial episodes, likely related to patients’ older age and more severe comorbidity. Our findings emphasize the need for prompt recognition of patients at risk to guide the choice of empiric treatment.

**Supplementary Information:**

The online version contains supplementary material available at 10.1007/s15010-025-02642-5.

## Introduction

Bloodstream infections (BSI) constitute a notable health burden worldwide. The incidence of BSIs has increased considerably over the past decades with reported rates ranging from 122 to 307/100,000 population [1–6]. Mortality of BSI remains high despite advances in antimicrobial treatment and intensive care.

Polymicrobial BSIs account for approximately 6–8.5% of all BSIs [1–3, 5, 7]. The occurrence of polymicrobial BSIs has increased which is probably related to an aging population with several comorbidities and immunocompromised conditions [4, 5, 7]. Polymicrobial BSIs are associated with higher case fatality than monomicrobial BSIs [4, 8, 9].

Population-based studies are considered the optimal design to evaluate the epidemiology of BSIs [3]. To our knowledge, no nationwide, population-based data are available concerning the incidence and outcome of polymicrobial BSIs. Few studies have been published on the outcome, characteristics, and causative microbes of polymicrobial BSIs, yet these studies mainly report results of a small cohort from a single or selected hospitals [9–12].

In our previous nationwide population-based study, we observed a substantial, twofold increase in the incidence of all BSI in Finland during 2004–2018 and furthermore, the proportion of polymicrobial BSIs rose from 6.5 to 7.1% [5]. In the current study, we utilized the same laboratory-based surveillance data linked to other registers to explore polymicrobial BSIs in Finland during 2004–2018. Our objective was to analyze patient characteristics, causative pathogens, and temporal trends of the incidence and outcome of polymicrobial BSIs. Moreover, our data allows a comprehensive comparison of polymicrobial and monomicrobial BSIs. Prompt recognition of polymicrobial etiology is crucial as the empiric antimicrobial therapy typically requires a more broad-spectrum coverage for effective treatment.

## Materials and methods

### Study setting and population

In Finland (population: 5.2 million in 2004, 5.5 million in 2018 [13]) all clinical microbiology laboratories notify all bacterial and fungal isolates from blood samples, i.e. BSI, to the National Infectious Disease Register (NIDR). The notifications are reported electronically and include the following information: specimen date, pathogen, susceptibility data for certain microbes, patient´s date of birth, sex, place of residence, and national identity code. Multiple notifications of the same pathogen containing the same identity code, i.e. referring to the same person, are merged into a case if they occur within 3 months of each other.

In this retrospective cohort study, we used NIDR data to identify all polymicrobial BSIs in Finland during 2004–2018; duplicate notifications (*n* = 155 BSIs) and BSIs with invalid identity codes (*n* = 587 BSIs) were excluded from the study (flow chart of the data in [5]). Information on the hospitalisation of the patient, including origin of the infection, i.e. community-acquired BSI (CA-BSI) vs. healthcare-associated BSI (HA-BSI), and current and prior (within 1 year) diagnosis codes were obtained by linkage to the National Hospital Discharge Register (HILMO).

### Definitions

Polymicrobial BSI was defined as isolation of more than one bacterial or fungal species in blood cultures within 2 days [14]. BSI was classified as healthcare-associated if the first blood culture was obtained more than 2 days after admission to hospital or within 2 days of discharge, and as community-acquired otherwise [15]. Patients with a BSI who were transferred from other healthcare facilities, including nursing homes, were also classified as having a HA-BSI. Comorbid illness was defined by using a validated algorithm for the Charlson comorbidity index (CCI) based on the International Classification of Diseases, 10th revision [16, 17]. Three levels of comorbidity were defined by the CCI scores: low (score 0), medium (score 1–2) and high (score > 2) [18]. The case fatality rate at 2, 7, 30 or 90 days after withdrawal of the first blood specimen with a positive culture was determined from the Population Information System by linkage with the identity code.

### Multidrug-resistant pathogens

The interpretation of susceptibility data of causative pathogens was performed in the clinical microbiology laboratories by using the CLSI standard for samples collected before year 2011 and, afterwards, according to the EUCAST clinical breakpoints [19]. The following bacteria with susceptibility data notified to the NIDR were defined as multidrug-resistant (MDR) pathogens: methicillin-resistant *Staphylococcus aureus* (MRSA) (starting year 1995), vancomycin-resistant *Enterococcus* (VRE) (1995), extended-spectrum beta-lactamase-producing (ESBL) *Escherichia coli* and *Klebsiella pneumoniae* (2008), and carbapenem-resistant Enterobacterales (CRE) (2015). ESBL-*E. coli* and ESBL-*K. pneumoniae* were defined as resistant or intermediately susceptible to third-generation cephalosporins, and CRE as *E. coli*,* K. pneumoniae* and *Enterobacter* sp. resistant or intermediately susceptible to carbapenems.

### Analyses and statistics

Population data from Statistics Finland [13] were used as denominators to calculate age- and gender-specific incidence rates of BSIs. Average annual incidence rates were determined according to the total number of episodes and population during 2004–2018. Poisson regression model, or negative binomial regression model in case of overdispersion, was applied to compare the observed trends in annual incidence rates, and log-linear binomial regression model for case fatality proportions. Univariate analysis of categorical variables was done with the Chi-squared test, using Yates’s correction or Fisher’s exact test, as appropriate. The differences in distributions between continuous variables were tested by the Kruskal-Wallis test. Data was analysed using SPSS Statistics version 29 (IBM) and Stata 18 (StataCorp).

## Results

In total, 173,715 BSIs were identified in the NIDR during 2004–2018; 11,347 (6.5%) were polymicrobial BSIs among 10,911 patients. Median age of the patients with polymicrobial BSI was 71 (range 0–102) (Table 1); 359 polymicrobial BSIs (3.2%) were reported in children aged < 16 years, including 171 BSIs (1.5%) in infants < 1 year of age. CA-BSIs represented 65.3% and HA-BSIs 34.7% of polymicrobial BSIs. One-fourth of the patients with polymicrobial BSI had a high CCI (score > 2) indicating severe comorbid conditions. During 2004–2018, the percentage of patients with polymicrobial BSI aged ≥ 70 years rose from 46.9 to 59.6%, and those with high CCI from 18.1 to 27.0%. Furthermore, the proportion of polymicrobial CA-BSIs increased from 60.2 to 75.8%, whereas HA-BSIs decreased from 39.8 to 24.2%.

**Table 1:** A comparison of characteristics and underlying comorbidities between polymicrobial and monomicrobial bloodstream infections, Finland, 2004–2018

Characteristics	All BSIs (*n* = 173,715)	Polymicrobial BSIs (*n* = 11,347)	Monomicrobial BSIs (*n* = 162,368)	*p* value
	No. (%)	No. (%)	No. (%)	
Gender				
Male	90,231 (51.9)	6,637 (58.5)	83,594 (51.5)	< 0.001
Female	83,484 (48.1)	4,710 (41.5)	78,774 (48.5)	
Age (years)				
Median (range)	70 (0–110)	71 (0–102)	70 (0–110)	
Age group (years)				
<20	8,943 (5.1)	434 (3.8)	8,509 (5.2)	< 0.001
20–69	76,062 (43.8)	4,940 (43.5)	71,122 (43.8)	
≥70	88,710 (51.1)	5,973 (52.6)	82,737 (51.0)	
Charlson comorbidity index				
Median (range)	1 (0–15)	1 (0–14)	1 (0–15)	
Score 0	70,328 (40.5)	4,045 (35.6)	66,283 (40.8)	< 0.001
1–2	66,364 (38.2)	4,472 (39.4)	61,892 (38.1)	
>2	37,023 (21.3)	2,830 (24.9)	34,193 (21.1)	
Origin of infection				
Healthcare-associated BSI	50,483 (29.1)	3,935 (34.7)	46,548 (28.7)	< 0.001
Community-acquired BSI	123,232 (70.9)	7,412 (65.3)	115,820 (71.3)	
Outcome				
Fatal within 2 days	6,392 (3.7)	756 (6.7)	5,636 (3.5)	< 0.001
Fatal within 7 days	10,752 (6.2)	1,203 (10.6)	9,549 (5.9)	< 0.001
Fatal within 30 days	22,474 (12.9)	2,342 (20.6)	20,132 (12.4)	< 0.001
Fatal within 90 days	32,387 (18.6)	3,221 (28.4)	29,166 (18.0)	< 0.001

BSI, bloodstream infection

In comparison with monomicrobial BSIs, polymicrobial BSIs were more often noted among male patients (58.5% vs. 51.5%) (Table 1). The proportion of HA-BSIs and patients with high CCI were greater in polymicrobial than in monomicrobial BSIs (34.7% vs. 28.7%, and 24.9% vs. 21.1%, respectively). In addition, patients with polymicrobial BSI were older than those with monomicrobial episodes; the proportion of patients aged ≥ 70 years was 52.6% among polymicrobial BSIs and 51.0% among monomicrobial BSIs. Of underlying conditions evaluated according to CCI, malignancy, metastatic malignancy, and peptic ulcer disease were more common in patients with polymicrobial BSI than in those with monomicrobial BSI (Table 2). Of the patients with polymicrobial BSI, malignancy, metastatic malignancy, and peptic ulcer disease were more frequently observed in males (59.1%, 57.0% and 62.1% males, respectively) than in females.

**Table 2:** A comparison of underlying comorbidities according to Charlson comorbidity index between polymicrobial and monomicrobial bloodstream infections, Finland, 2004–2018

Underlying comorbidity	All BSIs (*n* = 173,715)^a^	Polymicrobial BSIs (*n* = 11,347)^b^	Monomicrobial BSIs (*n* = 162,368)^c^	*p* value
	No. (%)	No. (%)	No. (%)	
Myocardial infarction	9,567 (5.6)	647 (5.8)	8,920 (5.6)	0.349
Congestive heart failure	21,444 (12.6)	1,197 (10.8)	20,247 (12.8)	< 0.001
Peripheral vascular disease	9,833 (5.8)	659 (5.9)	9174 (5.8)	0.484
Cerebrovascular disease	12,971 (7.6)	873 (7.9)	12,098 (7.6)	0.343
Dementia	9,528 (5.6)	652 (5.9)	8,876 (5.6)	0.207
Chronic pulmonary disease	15,479 (9.1)	970 (8.8)	14,509 (9.1)	0.160
Rheumatic disease	6,279 (3.7)	353 (3.2)	5,926 (3.7)	0.003
Peptic ulcer disease	2,303 (1.4)	211 (1.9)	2,092 (1.3)	< 0.001
Mild liver disease	6,172 (3.6)	440 (4.0)	5,732 (3.6)	0.054
Diabetes without chronic complications	22,172 (13.1)	1,469 (13.3)	20,703 (13.1)	0.549
Diabetes with chronic complications	9,706 (5.7)	614 (5.5)	9,092 (5.7)	0.396
Hemiplegia or paraplegia	1,015 (0.6)	78 (0.7)	937 (0.6)	0.136
Renal disease	11,292 (6.7)	682 (6.2)	10,610 (6.7)	0.028
Malignancy	38,323 (22.6)	3,487 (31.5)	34,836 (22.0)	< 0.001
Moderate or severe liver disease	2,067 (1.2)	165 (1.5)	1,902 (1.2)	0.007
Metastatic malignancy	4,285 (2.5)	456 (4.1)	3,829 (2.4)	< 0.001
AIDS/HIV	134 (0.1)	9 (0.1)	125 (0.1)	0.931

^a^Data available in 169,666/173,715 BSIs. BSI, bloodstream infection

^b^Data available in 11,084/11,347 BSIs

^c^Data available in 158,582/162,368 BSIs

### Causative microbes

In total, 25,185 different microbes were identified in the 11,347 polymicrobial BSI episodes, of which 49.0% (12,342/25,185) were gram-negative bacteria, 49.0% (12,331/25,185) were gram-positive bacteria, 1.6% (408/25,185) were fungi, and 0.4% (104/25,185) unclassified bacteria. *E. coli* (15.8%), enterococci (11.7%), coagulase-negative staphylococci (CNS) (9.5%), and *Klebsiella* sp. (8.8%) were the most common causative pathogens of polymicrobial BSIs (Table 3). Compared to monomicrobial BSIs, polymicrobial BSIs were more often caused by enterococci (11.7% vs. 4.5%), *Klebsiella* sp. (8.8% vs. 5.6%) and *Bacteroides fragilis* (4.9% vs. 1.4%). On the other hand, *S. aureus* (4.7% vs. 13.7%), *E. coli* (15.8% vs. 30.9%) and beta-haemolytic streptococci (2.9% vs. 8.0%) were less frequently identified in polymicrobial BSIs.

**Table 3:** A comparison of causative pathogens between polymicrobial and monomicrobial bloodstream infections, Finland, 2004–2018

Causative pathogen	Polymicrobial BSIs (*n* = 11,347 BSIs)	Monomicrobial BSIs (*n* = 162,368 BSIs)
	No. (%)	No. (%)
*Escherichia coli*	3,976 (15.8)	50,188 (30.9)
Enterococci	2,951 (11.7)	7,348 (4.5)
Coagulase-negative staphylococci	2,401 (9.5)	14,114 (8.7)
*Klebsiella* sp	2,225 (8.8)	9,025 (5.6)
*Bacteroides fragilis*	1,244 (4.9)	2,290 (1.4)
*Staphylococcus aureus*	1,191 (4.7)	22,297 (13.7)
*Streptococcus anginosus*	1009 (4.0)	1,829 (1.1)
*Clostridium* sp	947 (3.8)	1,082 (0.7)
*Pseudomonas aeruginosa*	832 (3.3)	3,670 (2.3)
Beta-haemolytic streptococci	742 (2.9)	13,068 (8.0)
*Enterobacter* sp	729 (2.9)	3,097 (1.9)
Other bacteria	6,530 (25.9)	31,813 (19.6)
*Candida albicans*	211 (0.8)	1,601 (1.0)
Other fungi	197 (0.8)	946 (0.6)
All microbes	25,185^a^ (100)	162,368 (100)

^a^7 different microbes in 4 BSIs, 6 microbes in 12 BSIs, 5 microbes in 77 BSIs, 4 microbes in 309 BSIs, 3 microbes in 1,574 BSIs, and 2 microbes in 9,371 BSIs. BSI, bloodstream infection

Of the causative pathogens of polymicrobial BSIs, 4,338/25,185 (17.2%) were obligate anaerobic pathogens (44.0% gram-positive bacteria, 54.7% gram-negative, and 1.3% unknown), whereas among monomicrobial BSIs anaerobic microbes represented 4.3% of all microbes (6,930/162,368) (37.0% gram-positive bacteria, 62.2% gram-negative, and 0.8% unknown). At least one anaerobic microbe was noted in 16.3% (1,850/11,347) of polymicrobial BSI episodes. During 2004–2018, the proportion of anaerobic bacteria in polymicrobial BSIs rose from 14.9 to 19.5%, an average annual increase of 2.4% (95% CI 1.8–3.0%, *p* < 0.001). Altogether 0.8% (196/25,185) of the causative pathogens detected in polymicrobial BSIs were MDR microbes compared to 1.8% (2,909/162,368) in monomicrobial BSIs. The majority (63.3%) of the MDR microbes in polymicrobial BSIs were ESBL-*E. coli*.

### Incidence

The average annual incidence of polymicrobial BSIs was 14.1 episodes/100,000 population, with higher rates among males than among females (16.8/100,000 vs. 11.5/100,000). The rates were greater in male than in female patients in all age groups except among children aged < 10 years and infants < 1 year of age. In patients aged ≥ 60 years the incidence was twofold in males compared to females. The highest overall incidence rates of polymicrobial BSIs were noted among patients aged ≥ 80 years (87.6/100,000).

**Fig. 1:**
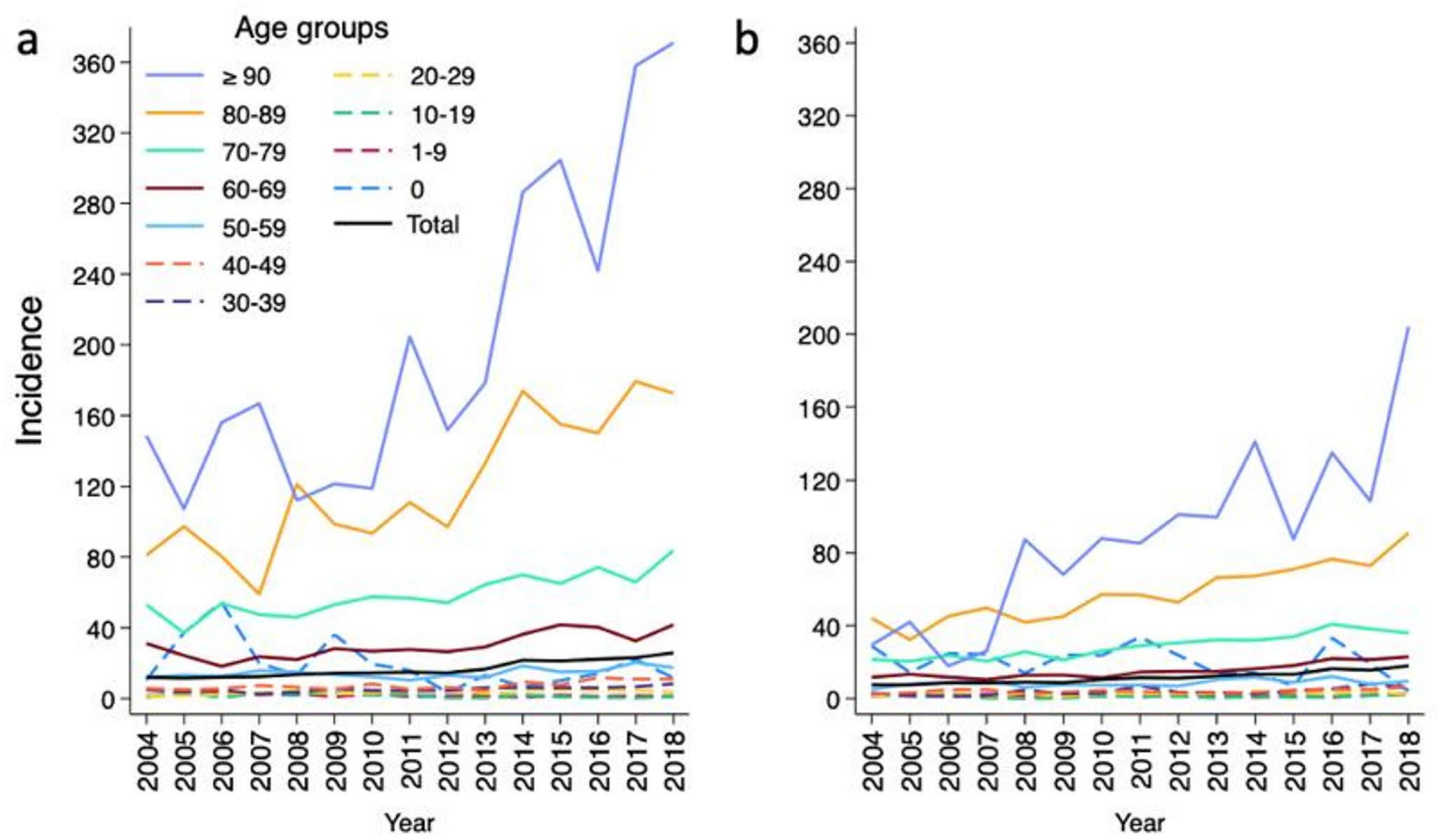
Annual incidence (episodes/100,000 population) of polymicrobial bloodstream infections, by gender and age group, Finland, 2004–2018. (**a**) Male patients; (**b**) female patients

During 2004–2018, the annual incidence of polymicrobial BSIs more than doubled, from 9.7 to 21.8/100,000 population, an average annual increase of 6.4% (95% CI 6.0–6.9%, *p* < 0.001) (Fig. 1). The rise was noted in both genders; in males from 12.0 to 25.8/100,000 population, an average annual increase of 6.3% (95% CI 5.7–6.9%, *p* < 0.001), and in females from 7.5 to 17.9/100,000 population, an average annual increase of 6.5% (95% CI 5.8–7.3%, *p* < 0.001). The incidence rose in all age groups ≥ 20 years of age with the most prominent increase observed among patients aged ≥ 90 years, from 54.3 to 245.1/100,000 population, an average annual increase of 11.3% (95% CI 9.1–13.5%, *p* < 0.001).

### Case fatality

Of all 11,347 polymicrobial BSI, 2,342 were fatal within 30 days and 3,221 within 90 days; the 30-day and 90-day case fatalities were higher for polymicrobial than for monomicrobial BSIs (20.6% vs. 12.4%, *p* < 0.001 and 28.4% vs. 18.0%, *p* < 0.001, respectively). The 30-day case fatality of polymicrobial BSIs was greater for female patients than for males (21.7% vs. 19.9%) and for HA-BSIs than for CA-BSIs (26.9% vs. 17.3%). The case fatality of polymicrobial BSIs rose with age; it was 3.5% (15/434) for patients aged < 20 years, 16.5% (815/4,940) for patients aged 20–69 years and 25.3% (1,512/5,973) for patients aged ≥ 70 years. Furthermore, the case fatality among patients with high CCI was three times greater compared to those with low CCI (32.0% vs. 10.7%).

**Fig. 2:**
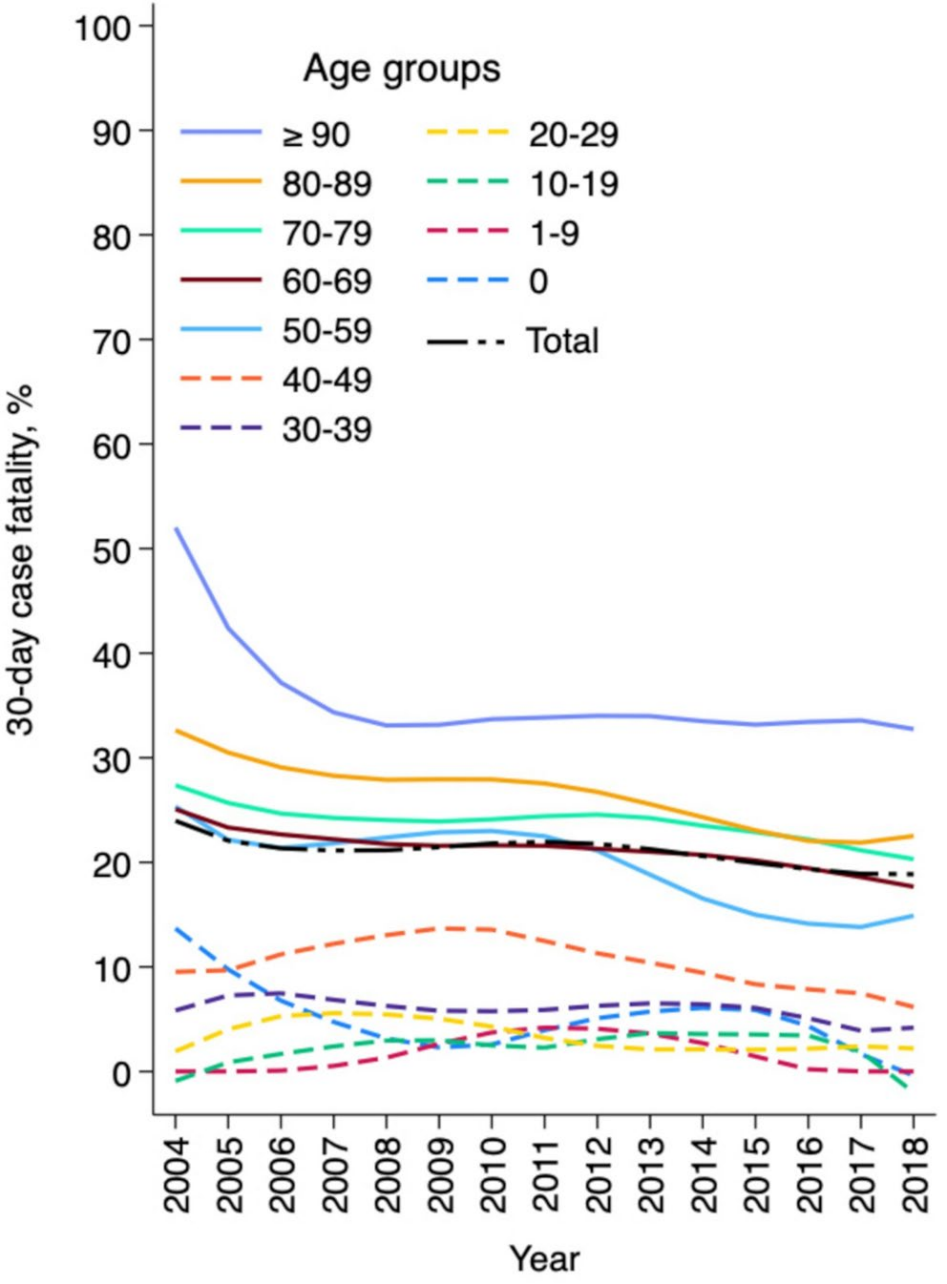
Annual 30-day case fatality of polymicrobial bloodstream infections and its smoothed values by lowess method, by age group, Finland, 2004–2018

During 2004–2018, the 30-day case fatality of polymicrobial BSIs declined from 25.2 to 20.8% (range by year, 16.2–25.2%) with an average annual reduction of 1.4% (95% CI 0.4–2.5%, *p* = 0.007) (Fig. 2). A descending trend in the 30-day case fatality was observed in both genders, in male patients from 25.2 to 19.9% (average annual reduction 1.7%, 95% CI 0.4–3.1%, *p* = 0.013) and in female patients from 25.4 to 22.0% (average annual reduction 1.0%, 95% CI -0.6–2.6%, *p* = 0.209). The 30-day case fatality of polymicrobial BSIs declined slightly in all age groups among patients aged ≥ 20 years.

Over half (51.4%) of the deaths following polymicrobial BSI occurred within 7 days (1,203/2,342) and one-third (32.3%) occurred within 2 days after the positive blood culture specimen (756/2,342). The 7-day and 2-day case fatalities of polymicrobial BSIs were 10.6% and 6.7%, respectively, both significantly higher than the 7-day and 2-day case fatalities of monomicrobial BSIs (5.9% and 3.5%, respectively). The patients with polymicrobial BSI who died within 2 days were older (63.1% vs. 51.9%, patients aged ≥ 70 years) and had more comorbidities (35.2% vs. 24.2%, patients with high CCI) than those who were alive at day 3 after the positive blood culture specimen. During 2004–2018, the 7-day case fatality of polymicrobial BSIs declined from 14.6 to 10.8% (average annual reduction 2.1%, 95% CI 0.8–3.5%, *p* = 0.002) and the 2-day case fatality from 9.5 to 6.2% (average annual reduction 2.3%, 95% CI 0.6–3.9%, *p* = 0.008).

## Discussion

Our nationwide population-based study comprising over 11,000 polymicrobial BSIs in Finland during fifteen consecutive years, provides a comprehensive outlook on the epidemiology of polymicrobial BSIs, along with an interesting comparison of monomicrobial and polymicrobial episodes. We demonstrated a notable, twofold rise in the incidence of polymicrobial BSIs during 2004–2018 with the steepest increase among elderly patients. Despite the growing proportion of aged patients and those with underlying illness, the 30-day case fatality of polymicrobial BSIs declined over time. Polymicrobial BSIs were more often caused by enterococci, *Klebsiella* sp., and anaerobic pathogens, compared to monomicrobial BSIs. Furthermore, the proportion of HA-BSIs, as well as comorbid and male patients was higher in polymicrobial BSIs than in monomicrobial episodes.

In our study, the average annual incidence of polymicrobial BSI was 14.1 episodes/100,000 population. The incidence rose considerably, from 9.7 to 21.8/100,000 population, with an average annual increase of 6.4% which is higher than the average annual increase of all BSIs (5.2%) in Finland during 2004–2018 [5]. To our knowledge, only a few regional population-based BSI studies have reported data on the incidence of polymicrobial BSIs during the past decades (Supplementary Table). A population-based study of all BSIs from Mid-Norway reported a rising incidence of fungal and polymicrobial BSIs analyzed together, from 9 to 11/100,000 person-years during 2002–2013 as well as an ascending trend in the incidence of all BSIs [4]. The proportion of polymicrobial and fungal BSIs combined rose from 10 to 13% together with an increasing incidence of all BSIs in an older population-based cohort study from Northern Denmark during 1992–2006 [18]. A population-based study on bacteremia from Funen County, Denmark, demonstrated a slight rise in the incidence of polymicrobial BSIs, from 15.4 to 15.6/100,000 person-years, yet unlike in other studies, the overall incidence of BSI declined during 2000–2008 [20]. Furthermore, in a multicenter cohort study of BSIs conducted in Andalucía, Spain, from 2006 to 2016, the proportion of polymicrobial BSIs of all BSIs increased from 6.2 to 8.7% concurrently with a rising incidence of all BSIs episodes; the proportion of both polymicrobial CA-BSIs and HA-BSIs rose over time [7]. Probable causes for the increasing occurrence of polymicrobial BSIs noted in previous studies include an aging population with multiple chronic diseases, such as malignancies and immunocompromised conditions.

We observed that polymicrobial BSIs occurred more often in males than in females which is in line with results from prior studies covering all BSIs, as well as with a study of polymicrobial BSIs in Japan [6, 20–23]. In our study, patients with polymicrobial BSI were older than those with monomicrobial BSIs, consistent with a cohort study from Minnesota, the United States, showing that the mean age of patients with polymicrobial BSI was higher than that of patients with gram-positive and gram-negative BSIs (71.5 vs. 61.8 and 63.1 years, respectively) [1]. In addition, patients with polymicrobial BSI in our study had more chronic diseases compared to patients with monomicrobial episodes, especially malignancies. Similarly, in an older cohort study from Colorado, the United States, the presence of nonhematologic malignancies and two or more underlying conditions were associated with the occurrence of polymicrobial BSI [24]. A Taiwanese case-control study also demonstrated that malignancies were more common among patients with polymicrobial BSI than among those with monomicrobial BSIs (32.1% vs. 21.1%) [10]. We noted a higher proportion of HA-BSIs in polymicrobial BSIs (34.7%) compared to monomicrobial BSIs (28.7%), in line with the study from Colorado, probably reflecting a higher burden of comorbid illness among patients with polymicrobial BSI [24]. Consistently, previous studies of all BSIs have presented higher proportion of polymicrobial BSIs among nosocomial and HA-BSIs compared with CA-BSIs [7, 20, 25].

In our study, polymicrobial BSIs were associated with considerably higher 30-day case fatality than monomicrobial BSIs, 20.6% vs. 12.4%. Comparably, in a population-based study of all BSIs from Mid-Norway, the 30-day case fatality for gram-negative BSI was 11.3%, for gram-positive BSI 18.5% and for polymicrobial and fungal BSIs together clearly higher, 31.8% [4] (Supplementary Table). In a case-control study from Taiwan, the 30-day fatality for polymicrobial BSIs was 30.4% compared with 11.6% for monomicrobial BSIs [10], and a Greek multicenter cohort of intensive care unit (ICU) and non-ICU patients presented 28-day case fatalities of 38.3% and 24.7% for polymicrobial and monomicrobial BSIs, respectively [12]. A cohort study from Taiwan during 2015–2016 reported worse outcome for polymicrobial BSIs than for monomicrobial episodes evaluated by 90-day mortality (44.9% vs. 33.6%), which is consistent with our results, yet higher rates observed in the Taiwanese study [9]. Polymicrobial etiology is also associated with late 1-year mortality as presented in a Canadian cohort study of community-onset BSIs, probably reflecting the severe underlying medical conditions of these patients [26].

In the present study, the case fatality of polymicrobial BSI was higher for females than for males (21.7% vs. 19.9%). In contrast, among monomicrobial BSIs, the case fatality was higher for males than for females (13.3% vs. 11.5%), which is in line with previous population-based studies covering all BSIs and with a cohort study of polymicrobial BSIs [4, 8, 24, 27]. In our study, of the patients with polymicrobial BSI who died within 30 days, female patients were older than males (71.6% vs. 59.1%, patients aged ≥ 70 years), which possibly contributed to the higher case fatality among female patients. Despite the growing proportion of aged and comorbid patients, we noted a decline in the case fatality of polymicrobial BSIs during 2004–2018 which might reflect progress in recognition of infection, antimicrobial treatment, and surgical source control.

A considerable proportion of polymicrobial BSI patients died early, within 2 days after the positive blood culture specimen resulting in a 2-day case fatality of 6.7%. Consistently, a population-based study of all CA-BSIs in Northern Denmark during 1992–1997, reported a 2-day case fatality of 10.7% for polymicrobial BSIs, which was higher than that of BSIs caused by *Streptococcus pneumoniae* (9.3%), *S. aureus* (9.0%), and *E. coli* (6.3%) [8]. In our previous study of all BSIs in Finland during 2004–2018 based on the same laboratory surveillance data as the present study, we demonstrated that polymicrobial etiology of BSI was an independent risk factor for fatal outcome in both outcome categories, death within 2 and 30 days, and both among patients with HA-BSIs and CA-BSIs [28].

The most common causative microbes of polymicrobial BSIs in our study were *E. coli*, enterococci, CNS and *Klebsiella* sp. Correspondingly, enterococci, *Klebsiella* sp., and *E. coli* were the most often identified causative pathogens of polymicrobial BSIs in a previous report from Japan [23]. Compared with monomicrobial BSIs, enterococci, *Klebsiella* sp. and anaerobic bacteria, which are all common bacteria of the lower gastrointestinal tract, were more frequently isolated in polymicrobial BSIs in the present study. In fact, we observed that the proportion of anaerobic bacteria as causative pathogens was markedly higher in polymicrobial than in monomicrobial BSIs (17.2% vs. 4.3%). Similarly, anaerobic microbes were detected in 18.9% of polymicrobial and in 4.0% of monomicrobial BSIs in a Taiwanese cohort study [9], and in 22.2% and 8.8% of polymicrobial and monomicrobial BSIs, respectively, in a study from the United States [24]. According to previous studies, intraabdominal infection represents the most common source of infection in polymicrobial BSIs [10, 23, 29] which is in accordance with the overrepresentation of anaerobic etiology in polymicrobial BSIs. Furthermore, the bowel, the biliary tract, and abscesses have been observed as more frequent sources of bacteremia in polymicrobial BSIs than in monomicrobial episodes [24]. Interestingly, in our study, the proportion of MDR microbes as causative agents was lower in polymicrobial BSIs than in monomicrobial BSIs (0.8% vs. 1.8%) indicating that MDR microbes did not play a substantial role in the outcome of polymicrobial BSIs. On the other hand, BSIs caused by anaerobic bacteria are generally associated with high fatality, up to 20%−27%, as demonstrated in previous studies [30–32], possibly contributing to the poorer outcome of polymicrobial BSIs compared to monomicrobial episodes.

There are several limitations in our study. First, as with other population-based studies utilizing retrospective surveillance and registry data, we had limited clinical information available. We did not have data on possible delays in recognition of the infection and initiation of the antimicrobial therapy, nor on the effectiveness of the chosen treatment. According to previous studies, the proportion of patients with polymicrobial BSI who receive inappropriate empiric antimicrobial treatment is high, up to 30 − 54% [10, 24, 33]. Moreover, we did not have information on the order and exact time frame (within 0, 1 or 2 days) in which the causative pathogens of polymicrobial BSIs were isolated in blood cultures, which might have influenced the appropriateness and delay of the administered antimicrobial treatment. The proportion of CNS as causative microbes of polymicrobial BSIs was considerable (9.5%) possibly indicating that some of these isolates represent contamination rather than true infection. However, the clinical microbiology laboratories in Finland exclude blood culture contaminations according to national guidelines, and isolates considered as contamination are not notified to the NIDR. No significant changes in blood culture methods have taken place in Finland during 2004–2018, yet it is possible that pathogen identification, for example, with the implementation of MALDI-TOF mass spectrometry, has improved during the study period and somewhat contributed to the increasing occurrence of polymicrobial BSIs.

Second, clinical features, such as the severity and focus of infection, surgical procedures, and potential behavioural risk factors of the patients were also unavailable. Moreover, we did not have data on the main cause of death of the patients who died within 30 days of the BSI, but it is likely that the infection was at least a contributing factor. Lastly, patients’ underlying medical conditions were assessed according to the CCI, which did not consider all chronic diseases, such as inflammatory bowel diseases, nor specific sites of malignancies. However, we did observe that the occurrence of inflammatory bowel diseases, biliary diseases and malignancies of digestive organs, for example, were more common among patients with polymicrobial BSI compared to those with monomicrobial BSI, by looking at specific ICD-10 diagnose codes (data not shown). A recent Australian population-based study demonstrated in fact that polymicrobial etiology was associated with higher risk for colorectal cancer-associated BSI [34].

Polymicrobial BSIs constitute a notable share of all BSIs and are associated with worse outcome than monomicrobial episodes. In our study, the incidence of polymicrobial BSIs increased considerably over time, most likely related to the aging of population and to patients’ rising burden of underlying illness and immunocompromised conditions. However, the case fatality of polymicrobial BSIs declined possibly indicating advancements in recognition and treatment of polymicrobial BSIs. Anaerobic microbes were substantially more common causative agents in polymicrobial BSIs than in monomicrobial BSIs. The empiric antimicrobial therapy of polymicrobial BSIs poses a challenge for clinicians, comparable to that of infections caused by MDR pathogens, as effective treatment may require broad-spectrum coverage. Further research for detailed data on clinical features including focus of infection and on patients’ predisposing underlying conditions and other risk factors is needed for early identification and guidance of empiric antimicrobial therapy of polymicrobial BSIs.

## Supplementary Information

Below is the link to the electronic supplementary material.

Supplementary Material 1

## Data Availability

No datasets were generated or analysed during the current study.
