# Supplementary material for: Characteristics, incidence and outcome of polymicrobial bloodstream infections: a nationwide population-based study, Finland, 2004–2018
Source: Infection. 2025 Sep 25;54(1):169–77. doi: 10.1007/s15010-025-02642-5 (PMC12864200; doi:10.1007/s15010-025-02642-5)
Supplement: Supplementary file 1 — Supplementary Material 1 [file 15010_2025_2642_MOESM1_ESM.docx]

**Supplementary Table** The incidence and outcome of polymicrobial bloodstream infections reported in studies published after 2000

|  | **Area, country** | **Time period** | **Setting** | **Catchment population** | **Proportion of polymicrobial BSI; time trend** | **Annual incidence of polymicrobial BSI (episodes per 100,000 population)** | | **30-day case fatality (%)** | **Comment** |
| --- | --- | --- | --- | --- | --- | --- | --- | --- | --- |
|  |  |  |  |  |  | **Average** | **Time trend** |  |  |
| **Population-based studies** | North Jutland, Denmark [8] | 1992–1997 | Regional; CA-BSIs | ~490,000 |  |  |  | 23.3% | 2-day case fatality of polymicrobial BSI, 10.7% |
|  | North Denmark Region [18] | 1992–2006 | Regional, hospitalized patients | ~500,000 | 10%–13% |  |  |  |  |
|  | Funen County, Denmark [20] | 2000–2008 | Regional; all BSIs | ~390,000 |  |  | 15.4–15.6 |  |  |
|  | Mid-Norway [4] | 2002–2013 | Regional; all BSIs | ~76,000 |  | 10 | 9–11 | 31.8% | Polymicrobial and fungal BSIs analyzed together |
| **Other studies** | Taiwan [10] | 2005–2007 | Case-control study, hospital emergency department |  |  |  |  | 30.4% |  |
|  | Greece [12] | 2007–2011 | Multicenter cohort study, ICU and non-ICU patients |  |  |  |  | 38.3% (28-day case fatality) |  |
|  | Taiwan [9] | 2015–2016 | Cohort study; two tertiary emergency departments |  |  |  |  | 44.9% (90-day case fatality) |  |
|  | Andalucía, Spain [7] | 2006 and 2016 | Multicenter cohort study |  | 6.2%–8.7% |  |  |  |  |

BSI, bloodstream infection

CA-BSI, community-acquired bloodstream infection

ICU, intensive care unit
